# Economic evaluation study (CHEER-compliant): Cost-effectiveness analysis of RAS screening for treatment of metastatic colorectal cancer based on the CALGB 80405 trial

**DOI:** 10.1097/MD.0000000000003762

**Published:** 2016-07-08

**Authors:** Jing Zhou, Rongce Zhao, Feng Wen, Pengfei Zhang, Ruilei Tang, Hongdou Chen, Jian Zhang, Qiu Li

**Affiliations:** a Department of Medical Oncology, Cancer Center, State Key Laboratory of Biotherapy; b Division of Liver Transplantation, Department of Liver Surgery, West China Hospital, Sichuan University, China.

**Keywords:** Bevacizumab, Cetuximab, cost-effectiveness, metastatic colorectal cancer, RAS

## Abstract

Cetuximab (Cetux)/Bevacizumab (Bev) treatments have shown considerably survival benefits for patients with metastatic colorectal cancer (mCRC) in the last decade. But they are costly. Currently, no data is available on the health economic implications of testing for extended RAS wild-type (wt) prior to Cetux/Bev treatments of patients with mCRC. This paper aimed to evaluate the cost-effectiveness of predictive testing for extended RAS-wt status in mCRC in the context of targeting the use of Cetux/Bev.

Markov model 1 was conducted to provide evidence evaluating the cost-effectiveness of predictive testing for KRAS-wt or extended RAS-wt status based on treatments of chemotherapy plus Cetux/Bev. Markov model 2 assessed the cost-effectiveness of FOLFOX plus Cetux/Bev or FOLFIRI plus Cetux/Bev in extended RAS-wt population. Primary base case data were identified from the CALGB 80405 trial and the literatures. Costs were estimated from West China Hospital, Sichuan University, China. Survival benefits were reported in quality-adjusted life-years (QALYs). The incremental cost-effectiveness ratio (ICER) was calculated.

In analysis 1, the cost per QALY was $88,394.09 for KRAS-Cetux, $80,797.82 for KRAS-Bev, $82,590.72 for RAS-Cetux, and $75,358.42 for RAS-Bev. The ICER for RAS-Cetux versus RAS-Bev was $420,700.50 per QALY gained. In analysis 2, the cost per QALY was $81,572.61, $80,856.50, $80,592.22, and $66,794.96 for FOLFOX-Cetux, FOLFOX-Bev, FOLFIRI-Cetux, and FOLFIRI-Bev, respectively. The analyses showed that the extended RAS-wt testing was less costly and more effective versus KRAS-wt testing before chemotherapy plus Cetux/Bev. Furthermore, FOLFIRI plus Bev was the most cost-effective strategy compared with others in extended RAS-wt population.

It was economically favorable to identify patients with extended RAS-wt status. Furthermore, FOLFIRI plus Bev was the preferred strategy in extended RAS-wt patients.

## 1 Introduction

More patients have the potential cure for metastatic colorectal cancer (mCRC) with the developments in surgery and chemo-biologic therapy in the last decade.^[1–3]^ Fortunately, novel targeted therapy has contributed to the recent progress of treating mCRC. Bevacizumab (Bev) is a humanized monoclonal antibody that inhibits the vascular endothelial growth factor (VEGF), a key mediator in tumor angiogenesis.^[4]^ Several meta-analyses have concluded that the use of Bev in first-line therapy for mCRC shows a benefit in overall survival (OS), progression-free survival (PFS), and response rates.^[5–8]^ Cetuximab (Cetux) is a chimeric monoclonal antibody that directly inhibited the downstream signaling pathways of epidermal growth factor receptor (EGFR). Recently, a meta-analysis of 14 randomized controlled trials concluded that the use of EGFR inhibitors in patients with KRAS (exon 2/codons 12 and 13) - wild-type (wt) mCRC has a clear survival benefit.^[9]^

Notably, the FIRE-3 trial compared the efficacy of FOLFIRI (5-fluorouracil [5-Fu], leucovorin, irinotecan) plus Cetux with FOLFIRI plus Bev in first-line therapy for KRAS-wt mCRC. A statistically significant improvement in OS was reported in the Cetux group. Furthermore, a marked OS advantage was noted for expanded RAS (exon 2, 3, and 4 of KRAS and NRAS) -wt patients in the Cetux group versus the Bev group.^[10]^ This is in line with the OS difference in the RAS-wt population of the CRYSTAL and OPUS trials, as well as for panitumumab (a human monoclonal antibody directed against EGFR) in a retrospective analysis of the PEAK and PRIME trials.^[11–15]^

Remarkably, the randomized, open-label, multicenter, phase III CALGB 80405 study compared first-line Cetux/Bev in combination with FOLFOX (5-Fu, leucovorin, oxaliplatin)/FOLFIRI. This trial did not meet its primary endpoint of OS (29.9 vs 29.0 months; HR 0.92; *P* = 0.34) between treatment groups in an initial analysis of the KRAS-wt population.^[16]^ In the expanded RAS-wt population, the median OS was pushed beyond 30 months, and there was higher objective response rate achieved in the Cetux group (68.6% vs 53.6%; *P* < 0.01). However, there was no significant difference between the Cetux and Bev in combination with chemotherapy in OS (32.0 vs 31.2 months; HR 0.90; *P* = 0.40) or PFS (11.4 vs 11.3 months; HR 1.1; *P* = 0.31). For the FOLFOX plus Cetux/Bev treatments in expanded RAS-wt patients, the OS was longer in the Cetux group than in the Bev group (32.5 vs 29.0 months; HR 0.86; *P* = 0.20). By contrast, the OS advantage in the expanded RAS-wt population was seen in favor of FOLFIRI plus Bev over FOLFIRI plus Cetux (32.0 vs 35.2; HR 1.1; *P* = 0.7).^[17]^

Besides, the PEAK and FIRE-3 trials retrospective subset analyses concluded that the PFS in RAS- mutant patients was significantly worse in receiving chemotherapy plus anti-EGFR therapy over chemotherapy plus Bev or chemotherapy alone. These results show that anti-EGFR therapy may even have a detrimental effect in RAS-mutant population.^[10,15]^ Overall, all the data emphasize the importance of extended RAS analysis in relation to the efficacy of anti-EGFR therapy. The National Comprehensive Cancer Network (NCCN) recommends that RAS mutation status should be determined and any known RAS mutation should not be treated with either Cetux or Bev.^[18]^

RAS mutation testing helps selecting the optimal treatment that patients would most benefit from. Additional costs of the novel predictive testing have to be balanced against cost savings associated with avoiding treatments for KRAS-wt patients who will not respond to Cetux/Bev therapy. Given the clinical efficacy data of extended RAS mutation testing for mCRC treatment, there was an interest in the economic analysis of RAS-wt screening. This paper aims to evaluate the cost-effectiveness of predictive testing for KRAS-wt or extended RAS-wt status in mCRC in the context of targeting the use of Cetux/Bev from a Chinese health care system perspective.

## 2 Methods

### 2.1 Patients and treatment regimens

The clinical information for the analyses was derived from the CALGB 80405 trial. The trial included patients with histologically confirmed and untreated KRAS-wt mCRC previously. They had a good performance status (Eastern Cooperative Oncology Group [ECOG] performance score of 0 or 1) and adequate organ functions. Patients received treatments according to physician-selected chemotherapy (FOLFOX or FOLFIRI) and were randomized to Cetux (initial dose 400 mg per m^2^ of body-surface area [BSA] and 250 mg per m^2^ of BSA weekly thereafter) or Bev (5 mg per kg of bodyweight) on each 14-day cycle. Treatments continued until disease progression or there was an unacceptable level of adverse events (AEs).

### 2.2 Model structure

We performed the cost-effectiveness analysis by using the Markov state transition model (TreeAge Software, Williamstown, MA). The study was approved by the Research Ethics Committees of West China Hospital, Sichuan University. The study does not involve patient consent, because clinical data were primary based on the CALGB 80405 trial, and costs estimated from West China Hospital, Sichuan University, China. Two analyses were conducted:

1. Analysis 1 was to provide evidence evaluating the cost-effectiveness of predictive testing for KRAS-wt or extended RAS-wt status based on treatments of chemotherapy (FOLFOX/FOLFIRI) plus Cetux/Bev.
2. Analysis 2 assessed the cost-effectiveness of FOLFOX plus Cetux/Bev or FOLFIRI plus Cetux/Bev in extended RAS-wt population.

**Figure 1:**
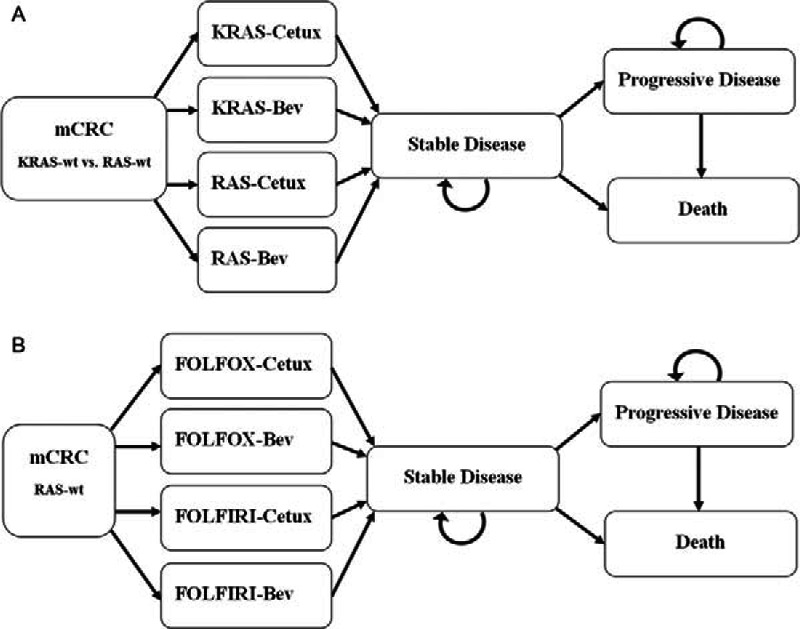
Overview of the Markov models. Simulation represents the transitions of the hypothetical cohorts through various health states from commencement of Stable Disease to Death. Bev = Bevacizumab, Cetux = Cetuximab, FOLFIRI = irinotecan, leucovorin, fluorouracil, FOLFOX = oxaliplatin, leucovorin, fluorouracil, mCRC = metastatic colorectal cancer.

Four strategies (Fig. 1A) were constructed in analysis 1: Chemotherapy plus Cetux in KRAS-wt patients (KRAS-Cetux), Chemotherapy plus Bev in KRAS-wt patients (KRAS-Bev), Chemotherapy plus Cetux in extended RAS-wt patients (RAS-Cetux), and Chemotherapy plus Bev in extended RAS-wt patients (RAS-Bev). In addition, analysis 2 constructed the following strategies (Fig. 1B): FOLFOX plus Cetux (FOLFOX-Cetux), FOLFOX plus Bev (FOLFOX-Bev), FOLFIRI plus Cetux (FOLFIRI-Cetux), and FOLFIRI plus Bev (FOLFIRI-Bev).

Our analyses were performed from the perspective of an estimated healthcare payer. The hypothetical cohort of patients proceeded from commencement of stable disease to death. The Markov structure was comprised of 3 mutually exclusive states: stable disease, progressive disease, and death. Patients began in the stable disease state, and they could reside in one of the stable disease states, move to one of the progressive disease states, or move to the death state during each model cycle.

Treatment effectiveness was summarized in terms of quality-adjusted life-years (QALYs). We compared the incremental cost-effectiveness ratio (ICER) among the strategies in analyses 1 and 2. The model cycle length was 1 month, and the time horizon chosen for the current analyses was a lifetime. Costs and benefits in our study were discounted at 3% annually.^[19]^

### 2.3 Base-case data

According to the CALGB 80405 trial, the median OS were 29.9, 29.0, 32.0, and 31.2 months in the KRAS-Cetux group, KRAS-Bev group, RAS-Cetux group, and RAS-Bev group, respectively. The median PFS was 10.4, 10.8, 11.4, and 11.3 months in the KRAS-Cetux group, KRAS-Bev group, RAS-Cetux group, and RAS-Bev group, respectively.^[16,17]^ Based on the transition probabilities estimated from PFS and OS, patients could switch to a different state at the end of each cycle in the Markov models. Monthly transition probabilities of health states were estimated as follows: *P* = 1– (0.5) [1/median time to event], which was derived from equations: *P* = 1 – e^–R^ and R=– ln[0.5]/(time to event/number of treatment cycles).^[20]^ The key input parameters are listed in Table 1.

**Table 1:**
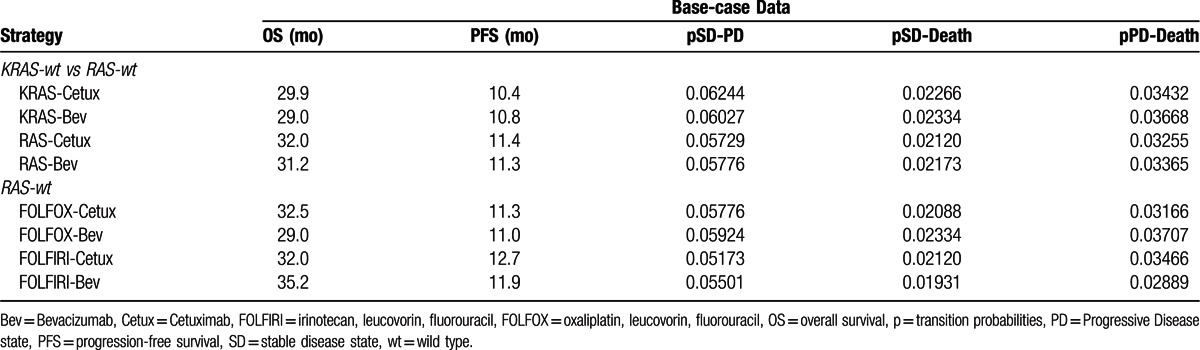
Clinical efficiencies and transition probabilities.

### 2.4 Costs

Costs in the present study consisted of direct medicine costs and indirect costs. Direct costs included fees for chemotherapeutic drugs, laboratory or imaging evaluation prior treatments, and chemotherapy administration (hospitalization and venous access). AEs-related costs and societal perspective costs (travel fees and absenteeism fees) constituted the indirect costs. The grades of AEs were defined according to the Common Terminology Criteria for Adverse Events (version 3.0). The frequencies of grades 3/4 AEs obtained from the trial were used to calculate the AEs-related costs. Data were based on a report on CALGB 80405 in 2014 ASCO Annual meeting.^[16]^ The incidences of total grade 3 and grade 4 AEs were 52% and 12.4% for the Bev group, 54% and 13.7% for the Cetux group. The primary grades 3/4 AEs were hematologic (26.6% for Bev, 27.4% for Cetux), neuropathy (14% for Bev, 12% for Cetux), rash (none for Bev, 7% for Cetux), diarrhea (8% for Bev, 11% for Cetux), hypertension (7% for Bev, 1% for Cetux), and gastrointestinal events (2% for Bev, 0.5% for Cetux). What needs illustration is that due to the absence of more details about hematologic events and the majority AEs-related patients had grade 3 hematologic AEs, we calculated the costs of hematologic events mainly based on grades 3 events. Moreover, the incidences of hematologic AEs used in our analysis are very close with the grades 3/4 hematotoxicity reported in the FIRE-3 study,^[10]^ which are 21% for the Bev group and 25% for the Cetux group. The travel fees were identical among the groups, which were $8.0 per patient each time based on the taxi fare in Sichuan, China. The cost for absenteeism every day was $18.94 according to the median monthly salary in Sichuan, China.^[21,22]^

An average BSA of 1.60 m^2^ and a mean body-weight of 58 kg were assumed.^[21]^ The cost of the drugs was on the basis of actual dose required per patient. A report on CALGB 80405 showed that 88% of patients received the subsequent therapies.^[16]^ However, the detailed information concerning the subsequent therapies has not yet been reported. We assumed that patients in CALGB 80405 received the subsequent therapies based on the NCCN practice guidelines for colon cancer,^[18]^ and the percentage of patients received the subsequent therapies from the Cetux group was comparable to that from the Bev group. Therefore, a weighted cost based on FOLFIRI ± Cetux/Bev/Panitumumab, Irinotecan ± Cetux/Bev/Panitumumab, FOLFOX/CapeOX ± Bev, Cetux or Panitumumab monotherapy was assumed per cycle for the medical costs after disease progression. The additional fees (costs for monitoring, administration, and societal perspective) during progressive disease treatment weighted upon the first-line treatment costs. We assumed the additional costs were identical among these groups. All costs were expressed in dollars (USD) and an exchange rate of $1 = ¥6.15 was applied in our study (November 20th, 2014). Table 2 lists the sources of costs in our analyses.

**Table 2:**
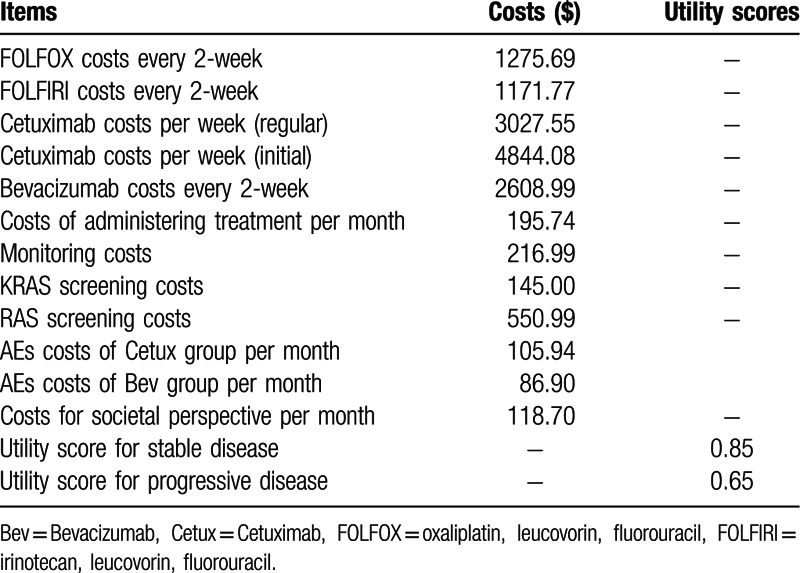
Costs and utility states.

### 2.5 Utilities

Utility score is preference weights that can be used to quantify the quality of life (QOL) in each state. QALYs for individuals were estimated based on the utility values. The values (Table 2) were derived from available literatures rather than the trial. Mean utility values were 0.85 in the stable disease state and 0.65 in the progressive disease states.^[23–25]^

### 2.6 Sensitivity analysis

One-way sensitivity analyses were performed to examine the robustness of the economic models, and the influence of the key input parameters on the results. We propagated low- and high-input-value estimates through the models and obtained the resulting range of ICER for each individual input. To assess the results of the base-case cost-effectiveness, the Markov model was run as a Monte Carlo simulation of 10,000 individuals and 1000 trials to account for uncertainty strategies.

## 3 Results

### 3.1 Cost outcomes

In the present report, Cetux or Bev were restricted to patients who benefited most from therapy, the addition of monoclonal antibodies to chemotherapy increased costs considerably (initial $4,844.08 and regular $3,027.55 for Cetux per week, $2,608.99 for Bev every 2-week, $1,275.69 for FOLFOX and $1,171.77 for FOLFIRI every 2-week). The AEs-related costs per month were $105.94 for the Cetux group and $86.90 for the Bev group. The costs estimated for the strategies are summarized in Table 2. When we running the Markov models to the estimated time horizon, the total costs for each strategy were as follows (Table 3): in the Markov model 1, $159,993.30 for KRAS-Cetux, $141,396.19 for KRAS-Bev, $157,748.27 for RAS-Cetux and $140,920.25 for RAS-Bev. In the Markov model 2, $158,250.86 for FOLFOX-Cetux, $140,690.31 for FOLFOX-Bev, $152,319.29 for FOLFIRI-Cetux and $138,933.51 for FOLFIRI-Bev, and the total AEs-related costs in the Bev group were lower than that in the Cetux group ($895.75 for the Cetux group and $642.18 for the Bev group).

**Table 3:**
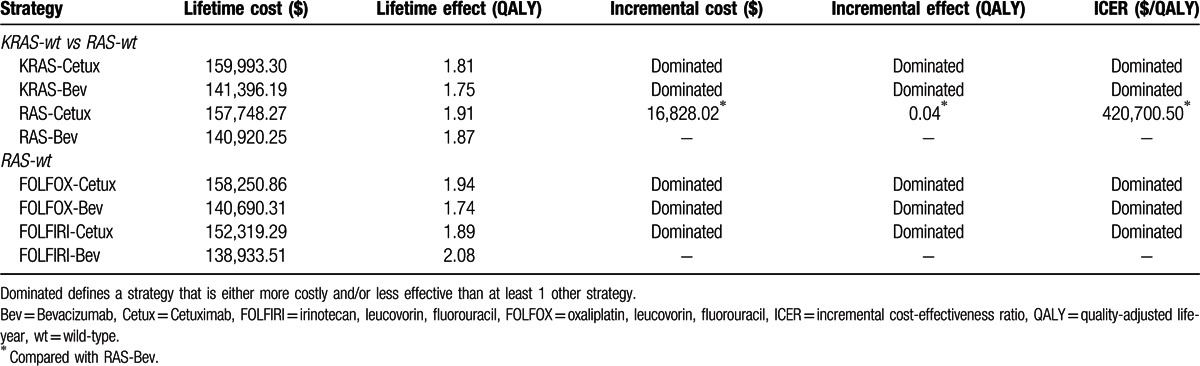
Results of base-case cost-effectiveness.

### 3.2 Effectiveness

According to the model base-case data analyses, the total obtained effectiveness of the treatments (with discounting) was listed in Table 3. The data in analysis 1 was 1.81 QALYs for KRAS-Cetux, 1.75 QALYs for KRAS-Bev, 1.91 QALYs for RAS-Cetux, and 1.87 for RAS-Bev. The effectiveness in analysis 2 was 1.94 QALYs for FOLFOX-Cetux, 1.74 QALYs for FOLFOX-Bev, 1.89 QALYs for FOLFIRI-Cetux, and 2.08 QALYs for FOLFIRI-Bev. Accordingly, the RAS-Cetux strategy led to the highest QALY result in the analysis 1, whereas the highest result was observed in the FOLFIRI-Bev strategy in the analysis 2.

### 3.3 Cost-effectiveness

**Figure 2:**
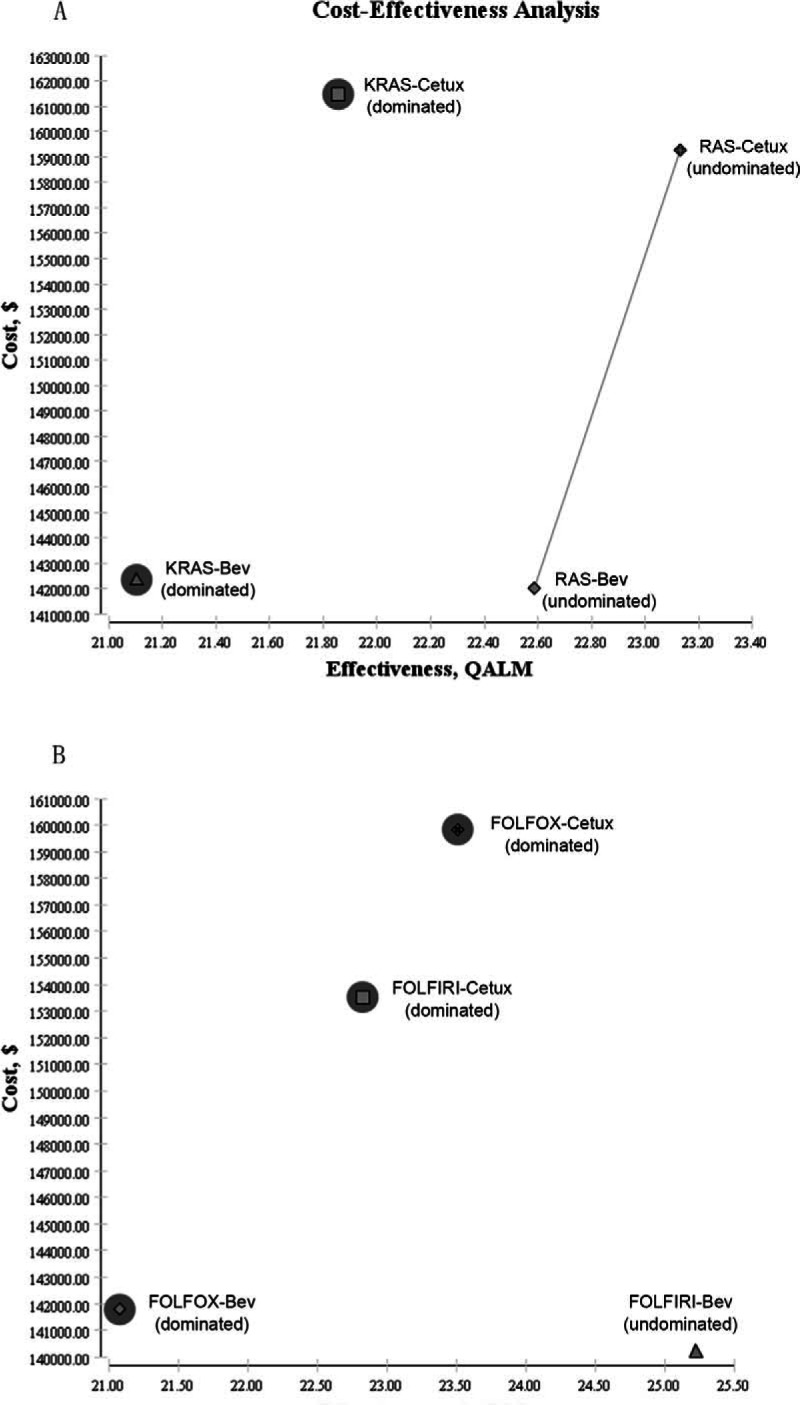
Cost-effectiveness plane. Bev = Bevacizumab, Cetux = Cetuximab, FOLFIRI = irinotecan, leucovorin, fluorouracil, FOLFOX = oxaliplatin, leucovorin, fluorouracil, QALM = quality-adjusted life-month. dominated, defines a strategy that is either more costly and/or less effective than at least one other strategy.

The mean costs and effectiveness gained by running the model as a Monte Carlo simulation were listed in Table 3. In analysis 1, the cost per QALY was $88,394.09 for KRAS-Cetux, $80,797.82 for KRAS-Bev, $82,590.72 for RAS-Cetux, and $75,358.42 for RAS-Bev. Accordingly, RAS-Bev was the least costly to gain a QALY. The RAS-Bev strategy was the least costly and the most effective compared with KRAS-Cetux or KRAS-Bev. RAS-Cetux significantly increased the cost of $16,828.02 to gain an additional 0.04 QALY versus RAS-Bev. In other words, the ICER for RAS-Cetux compared with RAS-Bev was $420,700.50 per QALY gained (Fig. 2A). In analysis 2, the cost per QALY was $81,572.61, $80,856.50, $80,592.22, and $66,794.96 for FOLFOX-Cetux, FOLFOX-Bev, FOLFIRI-Cetux, and FOLFIRI-Bev, respectively. FOLFIRI-Bev was the most cost-effective strategy compared with others (Fig. 2B).

### 3.4 Sensitivity analysis

**Figure 3:**
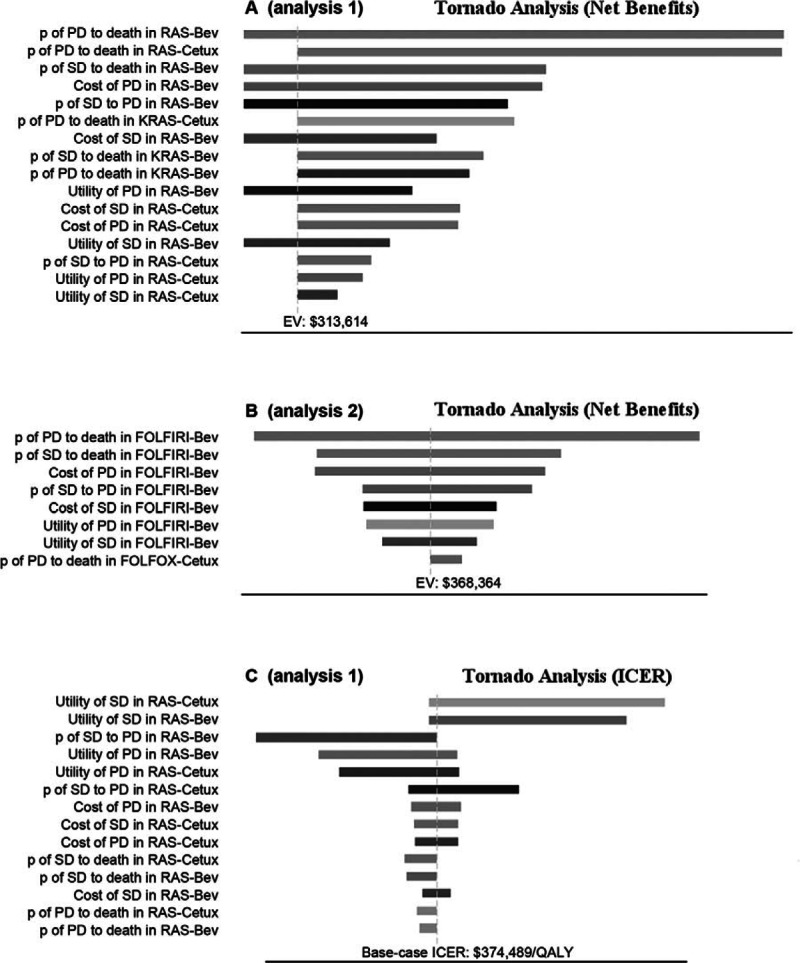
Tornado diagrams of 1-way sensitivity analyses. Bev = Bevacizumab, Cetux = Cetuximab, FOLFIRI = irinotecan, leucovorin, fluorouracil, FOLFOX = oxaliplatin, leucovorin, fluorouracil, ICER = incremental cost-effectiveness ratio, p = transition probability, PD = progressive disease, SD = stable disease, QALY = quality-adjusted life-years.

The results of 1-way sensitivity analyses were presented in tornado diagrams (Fig. 3). In analysis 1, varying the transition probability of progression to death in the RAS-Bev group had the strongest impact on the results (Fig. 3A). In analysis 2, the results were sensitive to changes in transition probability of progression to death in the FOLFIRI-Bev group (Fig. 3B). The first cost-effectiveness analysis provided evidence that it was economically favorable to identify patients with extended RAS-wt (RAS-Cetux and RAS-Bev were less costly and more effective vs KRAS-Cetux and KRAS-Bev). Thus, we tested the robustness of the ICER of RAS-Cetux versus RAS-Bev (Fig. 3C). It was sensitive to the utility of stable state in the RAS-Cetux group. When the value ranged from our baseline estimate of 0.85 up to 0.89, effectiveness in the RAS-Cetux group changed from 1.91 QALYs to 1.95 QALYs, and the ICER significantly decreased $181,069.06 per QALY gained. The impact of the transition probability of progression to death in the RAS-Bev group was minor to the results.

## 4 Discussion

Cetux/Bev treatments have considerably improved the OS of patients with mCRC in the last decade. But the monoclonal antibodies are costly. Nowadays, several cost-effectiveness analyses have shown that genetic mutations screening should be provided for anti-EGFR treatments in mCRC. The screening interventions are cost saving compared with providing anti-EGFR therapy to all mCRC patients. Patricia R. Blank et al^[26]^ recommended that it was economically favorable to identify patients with KRAS and BRAF mutation status, despite substantial costs of predictive testing. Recently, Behl et al^[27]^ drew a conclusion that screening for KRAS and BFAF mutation improved the cost-effectiveness of anti-EGFR therapy, but the ICER remains above the generally accepted threshold for acceptable cost effectiveness ratio of $100,000 per QALY. In a conference abstract, Shankaran et al^[28]^ estimated that it would realize $740 million in annual savings in the United States by providing KRAS testing to all 29,762 mCRC patients. Similarly, it has been estimated that KRAS testing to limit use of EGFR inhibitors to patients with KRAS-wt mCRC resulted in net savings of $7500 to $12,400 and of €3900 to €9600 per patient in the United States and Germany, respectively.^[29,30]^

As we all know, there is no economic analysis of predictive testing for KRAS-wt or extended RAS-wt in mCRC in the context of targeting the use of Cetux/Bev. We performed a head-to-head analysis based on the CALGB 80405 study. Notably, 2 analyses were conducted in the current report. When running the Markov models to the lifetime horizon, KRAS-Cetux cost 159,993.30 to yield 1.81 QALYs, KRAS-Bev cost 141,396.19 to yield 1.75 QALYs, RAS-Cetux cost 157,748.27 to yield 1.91 QALYs, and RAS-Bev cost 140,920.25 to yield 1.87 QALYs. The first Markov model showed that the RAS-Cetux and RAS-Bev were less costly and more effective compared with KRAS-Cetux or KRAS-Bev. This provided evidence that it was economically favorable to identify patients with extended RAS-wt. On the other hand, the ICER for RAS-Cetux versus RAS-Bev was $420,700.50 per QALY gained. In this case, the second Markov model was performed to assess the cost-effectiveness of FOLFOX plus Cetux/Bev or FOLFIRI plus Cetux/Bev in extended RAS-wt population. The cost per QALY was $81,572.61 for FOLFOX-Cetux, $80,856.50 for FOLFOX-Bev, $80,592.22 for FOLFIRI-Cetux, and $66,794.96 for FOLFIRI-Bev. In other words, FOLFIRI-Bev was the most cost-effective strategy compared with others in patients with extended RAS-wt status. One-way sensitivity analyses indicated that the transition probability of progression to death in RAS-Bev (analysis 1) and FOLFIRI-Bev (analysis 2) played a key role on the results. Moreover, the ICER of RAS-Cetux versus RAS-Bev was sensitive to the utility of stable state in the RAS-Cetux group.

The societal willingness-to-pay (WTP) thresholds vary between countries. Some used $20,000 per QALY as the WTP threshold,^[31]^ whereas the others suggested that the ICER less than $50,000 per QALY are cost-effective.^[32,33]^ We set the WTP threshold based on the guidelines of the World Health Organization (WHO). The threshold used in the study was $20,301, triple the per capita GDP of China.^[34]^ In the analysis 1, extended RAS-wt screening was cost-effective compared with KRAS-wt testing before treatment. Furthermore, the ICER of RAS-Cetux versus RAS-Bev ($420,700.50/QALY) was thought to be unacceptable at the WTP threshold of $20,301. That is to say, RAS-Bev (extended RAS-wt screening before chemotherapy plus Bev) was a cost-effective choice for patients with mCRC. On the other hand, as we detailed above, FOLFIRI-Bev was the most cost-effective strategy in extended RAS-wt patients.

Obviously, there are differences in survival outcomes between FIRE-3 and CALGB 80405 trials. The following reasons might illustrate the discrepancy between the 2 studies. First, chemotherapy backbones were heterogeneous between the 2 studies, and the sample size in CALGB 80405 trial is much smaller than that in FIRE-3 trial. Less than one-third of RAS-wt patients in CALGB 80405 trial received FOLFIRI chemotherapy, and in particular it was of physician choice. Second, subsequent therapies may serve as a major determinant of OS. Recently, a study reported that the percentage of patients received subsequent therapies in FIRE-3 trial was comparable to that in the CALGB 80405 trial.^[35]^ However, detailed information on CALGB 80405 concerning the analyzed population and the exact subsequent therapies has not yet been reported, which will potentially help to identify differences and similarities between the 2 studies. Third, a biologically-based difference in effectiveness that might exist on the sequence of Bev and Cetux been used. Lastly, the selected laboratory techniques for testing RAS state might partly account for discrepant OS between the 2 studies. To test the RAS state, BEAMing was chosen and the cut-off value was 1% for the CALGB 80405 trial, whereas pyrosequencing was selected and the cut-off value was 5% for the FIRE-3 trial. Moreover, only 55% of patients in the CALGB 80405 study tested the RAS state, due to the research spanned the last decade and there was not enough nor suitable specimens for RAS testing.

Admittedly, cost-effectiveness analysis should incorporate a prospective collection of costs and quality of life. The clinical data of our study were derived from CALGB 80405 trial. When we running the Markov models to the estimated time horizon, FOLFIRI-Bev was the most cost-effective strategy (achieved the most effectiveness with the least cost) in patients with extended RAS-wt status. Besides, the total costs of Cetux group were higher than that of Bev group in the KRAS-wt population, which are consistent with the fees reported in a cost study on CALGB 80405 in 2015 ASCO Annual meeting,^[36]^ demonstrating that our results are reliable in a manner. In all, it is reasonable for our conclusion that FOLFIRI with Bev is a preferred strategy in all RAS-wt population.

The data in our study should be interpreted in the context of its limitations. First, the cost data were derived from West China Hospital, Sichuan University, China. The sensitivity analyses showed that the results were robust to changes in some estimated parameters. Second, the analysis relied on the CALGB 80405 trial and literatures to provide estimates of base case data. For example, QOL adjustment is an important part of cost-effectiveness research, the reliance on a few published utility values in the literature is not ideal. Therefore, the inclusion of real-life data would strengthen the analyses. But these are probably not the relative cost-effective differences between strategies. Finally, although the QALYs derived from the present analyses may apply to other countries, the economic analysis results are not directly generalizable given differences in costs and practice patterns. More details should be studied to figure out the further cost-effectiveness of these strategies.

In conclusion, it is the first head-to-head cost-effectiveness study to evaluate predictive testing for extended RAS-wt in mCRC in the context of targeting Cetux/Bev treatment. The results demonstrate that it was economically favorable to identify patients with extended RAS-wt status. Furthermore, FOLFIRI plus Bev was the preferred strategy compared with other strategies in patients with extended RAS-wt, at a societal WTP threshold of $20,301 in a Chinese perspective. We believe our study will encourage physicians to make the optimal treatment choice. Future clinical trials should incorporate a prospective collection of costs and quality of life with limited healthcare resources. The new anticancer therapies should acquire the maximizing societal benefits and maintaining the sustainability of the country's healthcare system.
